# Bacteriophage-Derived Endolysins Applied as Potent Biocontrol Agents to Enhance Food Safety

**DOI:** 10.3390/microorganisms8050724

**Published:** 2020-05-13

**Authors:** Yoonjee Chang

**Affiliations:** Department of Food and Nutrition, Kookmin University, Seoul 02707, Korea; ychang@kookmin.ac.kr; Tel.: +82-2-910-4775

**Keywords:** endolysin, antimicrobial, biocontrol, disinfection, food safety

## Abstract

Endolysins, bacteriophage-encoded enzymes, have emerged as antibacterial agents that can be actively applied in food processing systems as food preservatives to control pathogens and ultimately enhance food safety. Endolysins break down bacterial peptidoglycan structures at the terminal step of the phage reproduction cycle to enable phage progeny release. In particular, endolysin treatment is a novel strategy for controlling antibiotic-resistant bacteria, which are a severe and increasingly frequent problem in the food industry. In addition, endolysins can eliminate biofilms on the surfaces of utensils. Furthermore, the cell wall-binding domain of endolysins can be used as a tool for rapidly detecting pathogens. Research to extend the use of endolysins toward Gram-negative bacteria is now being extensively conducted. This review summarizes the trends in endolysin research to date and discusses the future applications of these enzymes as novel food preservation tools in the field of food safety.

## 1. Introduction

Contamination of food by foodborne pathogens is a serious issue in the food industry; for example, contamination by *Staphylococcus aureus*, *Salmonella* spp., *Escherichia coli*, *Listeria monocytogenes*, and *Clostridium* spp. during food processing can threaten human health and lead to economic losses [1]. Therefore, it is recognized that novel strategies to control pathogenic bacteria in foods are urgently required.

Endolysins are bacteriophage (phage)-encoded peptidoglycan hydrolases that are synthesized at the end of the phage multiplication cycle; they lyse the host bacterial cell wall and release newly assembled bacteriophage virions [1]. Specifically, endolysins directly target various bonds in the bacterial cell wall peptidoglycan structure [2]. In this process, holin proteins assist their entry into the cytoplasmic membrane where endolysins lyse the bacterial cell wall [3]. In general, endolysins can act as exolysins in the Gram-positive bacterial peptidoglycan layer; however, they cannot degrade the bacterial outer membrane around Gram-negative bacterial cells [4]. Indeed, the outer membrane of Gram-negative bacteria effectively prevents access of the endolysin. Therefore, researchers have attempted to develop new methods for using endolysins against Gram-negative pathogens.

Endolysins from Gram-positive phages have a modular structure composed of enzymatically active domains (EADs) and a cell wall-binding domain (CBD) [5]. The EADs provide the actual enzymatic activity that cleaves the peptidoglycan structure, whereas the CBD recognizes and leads the endolysin to the specific cell wall-associated ligand molecules with high specificity.

The use of endolysins is considered to be safe as they do not create gene transduction issues or contribute to the emerging problem of resistant bacteria. Although there are concerns about the application of phages such as the emergence of phage-resistant bacteria and gene transduction [6], endolysins do not create such problems [7]; therefore, endolysins are promising biocontrol agents that could be applied in the field of food safety. Although many studies on the medical applications of endolysin have been published, studies of their use in the food industry have yet to be actively conducted [4,8,9]. Nevertheless, there an increasing interest from the food industry around the use of endolysins. Therefore, this review aims to provide an up-to-date overview of the use of endolysins and ideas for their use as control agents against foodborne pathogens. Both the fundamental questions about endolysins and their potential food applications are addressed.

## 2. Endolysins: Structure, Enzymatic Function, and Substrate Recognition

Endolysins are expressed during the late phase of gene expression in the double-stranded DNA phage lytic cycle [2]. After phage replication inside the bacterial host, phage progeny must be released by cell wall degradation. Endolysins participate in this step by weakening the bacterial cell wall and hydrolyzing the peptidoglycan of the host.

**Figure 1:**
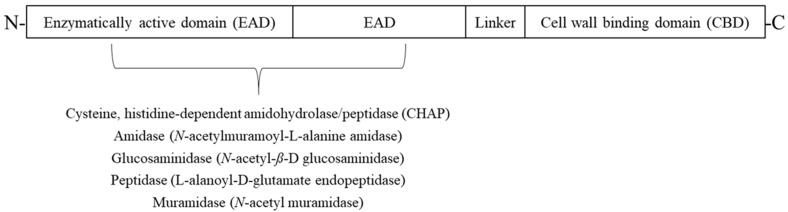
Schematic representation of the modular structure of phage-encoded peptidoglycan hydrolases. Most endolysins contain one or two enzymatically active domain (EAD) in N-terminal those cleave one of the bonds in the bacterial peptidoglycan, and one cell wall binding domain (CBD) involved in host bacterial recognition in C-terminal region. EAD and CBD are connected by a short linker.

In general, endolysins exist in a modular structure composed of at least two separated functional domains (Figure 1). Gram-positive endolysins have some EADs at the N-terminal end and a CBD at the C-terminal end, and they are connected with a short linker [2]. As they possess both EADs and a CBD, endolysins have both enzymatic hydrolysis and host bacteria substrate recognition functions, respectively. Specifically, the EAD participates in the cleavage of various bonds in the peptidoglycan of the bacterial cell wall, whereas the CBD recognizes and binds to the bacterial cell wall with high specificity. In contrast to Gram-positive endolysins, Gram-negative endolysins generally have a globular structure that only possesses EADs; they rarely show a modular structure [10]. The few Gram-negative endolysins that have a modular structure all have an inverted molecular structure relative to Gram-positive endolysins: the EADs are located at the C-terminal end, and the CBD is located at the N-terminal end (e.g., *Pseudomonas* endolysin KZ144 [11]). One endolysin, namely OBPgp279 from the *Pseudomonas putida* phage OBP, has been predicted to have two CBDs [12]. Interestingly, CBDs in Gram-positive endolysins show high host specificity and enhance the substrate affinity of the enzyme, whereas CBDs from Gram-negative endolysins show a broad binding spectrum [10].

Several types of EAD exist: amidases, glycosidases, and carboxy/endopeptidases. Glycosidases attack the linkages of the amino sugar moieties, whereas amidases and peptidases cleave the amide or peptide bonds of the cross-linking peptides and interpeptide bridges [2]. Endolysins show high specificity as they have a CBD that recognizes and binds to the substrate [13]; therefore, the EAD acts much efficiently when it coexists with the CBD as it leads the endolysin to the host cell membrane with high affinity [14]. Consequently, the endolysin can specifically target bacteria as CBD specifically binds to the host.

## 3. Mechanisms of Action of Endolysins against Gram-Positive Pathogens

**Figure 2:**
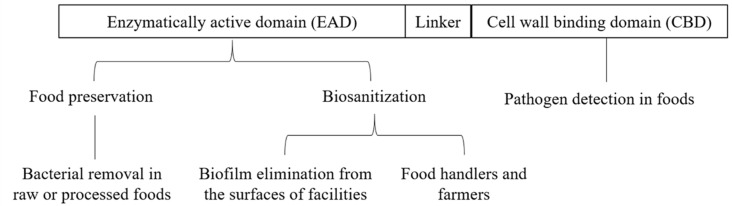
Applications of endolysins in food science fields for improving food safety. Enzymatically active domain (EAD) have been mainly used in food preservation and disinfection for enhancing food safety. Specifically, pathogens in raw materials or processed foods, and biofilms produced in surfaces of facilities in food industry can be removed by EAD or full endolysin [17]. Moreover, pathogens in food handlers and farmers can also be controlled by EAD or full endolysin [29]. Pathogen detection has mainly been done by the cell wall binding domain (CBD) [16].

Various applications of endolysins in food science fields are introduced in Figure 2. According to the bonds that endolysins target, they can be classified into five different classes [2]. The *N*-acetylmuramidases (lysozymes), *N*-acetylglucosaminidases, and transglycosylases target the sugar backbone moiety of peptidoglycan; endopeptidases attack the peptide moiety; and *N*-acetylmuramoyl-L-alanine amidases, which are predicted to lead the strongest damage in the peptidoglycan, cleaving the amide bond between N-acetylmuramic acid and L-alanine. Among the endolysins, muramidase, which is found in the *Pseudomonas aeruginosa* phage phiKZ gp144 lysin [15], is the rarest type; in contrast, amidases that hydrolyze the most conserved bonds in the peptidoglycan are most widely distributed [16,17,18,19]. Studies of endopeptidases have shown that *Listeria* endolysin Ply500, Ply118 [20], and some of the *Bacillus cereus* endolysins [21,22] possess L-alanyl-D-glutamate endopeptidases. Other, staphylococcal endolysin phi11 possess D-alanyl-glycyl endopeptidase and cleave within peptides that cross-link the cell wall [23].

Endolysins can act efficiently when they co-exist with holin [3]. Holin leads the endolysins to move toward their substrate [3]. During phage maturation in infected bacteria, endolysins accumulate in the cytoplasm. Subsequently, the holin proteins penetrate the cytoplasmic membrane and form holes that allow the endolysins to get close to, and attack, the peptidoglycan; this results in cell lysis and the release of progeny phages [3]. Until the bacterial cell loses its rigidity, endolysins destroy the peptidoglycan layer and disrupt internal osmotic pressure.

In general, Gram-positive phages have a holin–endolysin system, in which holin gives the endolysin access to the cytoplasmic membrane where it damages the bacterial cell wall [24]. A few phages contain signal peptides other than holin, which lead the proteins to secretory pathways [25]. Importantly, when Gram-positive endolysins are applied externally to bacterial cells, they can directly access the cell wall carbohydrates and peptidoglycan membrane from outside of the cells, thereby acting as antibacterial agents [10]. Moreover, a small amount of purified endolysin is sufficient to rapidly lyse Gram-positive bacterial cells within minutes or even seconds [21]. Therefore, researchers have targeted Gram-positive endolysins as biocontrol agents against various pathogens including *Streptococcus pneumonia*, *S. aureus*, *L. monocytogenes*, *Enterococcus faecalis*, and *Clostridium perfringens* [26,27,28].

## 4. Application of Endolysins against Gram-Negative Pathogens

In contrast to Gram-positive bacteria, Gram-negative cells are resistant to external endolysin treatment as they possess an outer membrane on their cell wall that prevents the interaction between the endolysins and peptidoglycan layer [10]. Although Gram-positive endolysins have been applied as biocontrol agents, recent studies have also reported methods to overcome the outer membrane barrier in order to lyse and kill Gram-negative bacteria [30,31,32].

The use of outer membrane-permeabilizing agents such as chelators is the most common strategy for increasing the effectiveness of Gram-negative endolysins as biocontrol agents. For example, chelators such as ethylenediaminetetraacetic acid (EDTA) and organic acids (citric and malic acids) have generally been used as outer membrane permeabilizers [30,31]. A specific example comes from the endolysin OBPgp279, which was reported to have bactericidal activity (approximately 1-log reduction in activity within 30 min) against *Salmonella* Typhimurium cells when used in combination with EDTA [33]. In addition, Oliveira et al. [32] demonstrated that the *Salmonella* endolysin Lys68 kills Gram-negative cells such as *Salmonella*, *Acinetobacter*, *Pseudomonas*, *Shigella*, *E. coli* O157:H7, *Cronobacter sakazakii*, *Pantoea*, *Enterobacter*, and *Proteus* when combined with citric or malic acid. In this study [32], the organic acid treatment led to a higher efficiency than an EDTA treatment: the authors showed a ~5-log CFU/mL bacterial cell reduction within 2 h when the endolysin was applied externally in slightly acidic conditions. However, the use of both EDTA and organic acids with endolysins remains problematic as EDTA is known to harm human cells and organic acids can inactivate the endolysin in acidic pH conditions [30]. In other studies, the combined treatment of endolysins with physical stressors such as high hydrostatic pressure also produced endolysin antibacterial effects [31]. Specifically, high hydrostatic pressure led to the transient permeabilization of the outer membrane and allowed the Gram-negative endolysin to access the substrate [31]. In another case, the *Cronobacter sakazakii* endolysin LysSs1 showed antibacterial activity against Gram-negative bacteria when the bacterial cells were pretreated with heat or chloroform to destabilize the integrity of the outer membrane [34].

To control Gram-negative bacteria with endolysins, researchers have developed the concept of fusing Gram-negative endolysins with membrane-penetrating peptides. Such genetically modified endolysins are known as Artilysins; they are composed of some endolysin domains, linker units and the hydrophobic/amphipathic peptides [35]. Through this strategy, pretreatment with membrane permeabilizers is not required as the Artilysins penetrate the outer membrane of Gram-negative cells naturally. However, advanced molecular biological techniques are required to select the optimal membrane-destabilizing peptides and construct genetically modified proteins. For example, one Artilysin comprised of the OBPgp279 endolysin and a polycationic nonapeptide was reported to kill >3-log CFU/mL Gram-negative bacterial cells within 30 min [35].

Although their lytic activity is not strong, some endolysins have been reported to kill Gram-negative bacteria naturally from the outside of bacterial cells. For example, the *Salmonella* phage SPN9CC endolysin was reported to kill about 2-log CFU/mL *E. coli* cells within 1 h of reaction [36]. The *Acinetobacter baumannii* endolysin LysAB2 and its modified derivatives have also been revealed to show antibacterial activities against Gram-negative bacteria (*Acinetobacter* and *E. coli*) without assistance [37]: amphipathic peptides containing basic amino acid residues potentially penetrate the negatively charged outer membrane; however, the activity is weak because the extent of penetration is limited. In a recent study, a novel lysis mechanism was reported regarding the *Salmonella* endolysin M4Lys that, when compared with other endolysins, was not dependent on either holin or the Sec pathway [38]. It was revealed to have a unique mosaic structure, and the C-terminal transmembrane domain had a critical role in M4Lys-mediated lysis in bacterial cell.

In a novel work on Gram-negative endolysins, the application of a liposome-mediated endolysin encapsulation system was developed [30]. Specifically, the *Salmonella* endolysin BSP16Lys was encapsulated into a cationic liposome system [30]. As liposomes are known to be able to penetrate bacterial membranes by membrane fusion, the BSPLys-encapsulated liposome produced a ~2.2-log CFU/mL reduction in *Salmonella* without treatment of a membrane permeabilizer [30]. The application of endolysins to kill Gram-negative pathogens is still in development as researchers increase their interest in finding novel strategies to control Gram-negative bacteria.

## 5. Food Safety Applications of Endolysins as Biocontrol Agents

The number of outbreaks of foodborne diseases is increasing, and the emergence of antibiotic-resistant bacteria is a problematic issue. Accordingly, many research groups have shown an interest in endolysins as alternative bactericidal agents to synthetic antimicrobials, including antibiotics.

Although phages are good biocontrol candidates, some problems exist when considering phages as antimicrobial agents for use in the food industry; these include the need to select a virulent phage to avoid transduction and the potential development of bacteria that are resistant to phages [39]. Therefore, endolysins have been considered as potential alternative biocontrol agents; indeed, endolysins have already been applied in food systems to prevent pathogen contaminations [17,23,40,41,42,43,44,45,46,47,48,49,50,51,52,53]. Purified endolysins can be directly added to foods. Furthermore, endolysins can be produced and secreted by fermenting bacteria such as *Lactococcus lactis* or *Lactobacillus* spp. Obviously, the safety and stability of endolysins must be considered ahead of their application to food products and in food processing facility systems. Therefore, studies exist in which the safety of the endolysins is reported; for example, after endolysin SAL-1, Cpl-1, and Pal injections, animals did not show mortality or signs of toxicity [54,55]. There are several studies of endolysins those introduced the potential for food applications (Table 1). 

The staphylococcal endolysin LysH5 was used in milk to control *S. aureus* for the first time; it showed a strong bactericidal activity with a reduction of about 8-log CFU/mL reported [56]. Related to this finding, LysH5 showed a synergistic bactericidal effect when it was used as co-treatment with nisin, which has different antibacterial mechanisms [41]. Thus, these antibacterial agents could be used as potent milk preservative candidates. The LysH5 endolysin also showed synergistic bactericidal effects with the phage vB_SauS-phiIPLA88 HydH5 virion-associated peptidoglycan hydrolase; however, this endolysin–hydrolase combination has yet to be applied to food [57]. These synergistic systems can be applied as hurdle technologies to enhance food safety with their low endolysin concentrations, low costs, and reduced risk of bacterial resistance. In a recent study, the *S. aureus*-targeting endolysin LysSA97 was purified and applied with essential oil to various foods including whole milk, skimmed milk, and lean beef [45]. As a result, both LysSA97 and carvacrol produced a synergistic antibacterial effect in all of the food systems tested. The strongest effect was observed in skimmed milk, which had less fat than other food items; thus, the antibacterial effect appeared to be influenced by the total lipid content of the foods [45]. LysSA11 endolysin, which also targets methicillin-resistant *S. aureus* (MRSA), was purified and applied to foods (milk and ham) and utensil systems (polypropylene plastic cutting boards and stainless steel knives) [17]. Results showed that LysSA11 produced a 2-log CFU/mL bacterial reduction in milk and a >3-log CFU/cm^3^ reduction in ham, both in refrigerator (4 °C) and room temperatures (25 °C) [17]. Additionally, LysSA11 killed MRSA with high efficiency on utensil surfaces: complete bacterial killing was observed after a 30 min treatment with a small quantity of the endolysin [17]. In another study, the *Listeria monocytogenes* endolysin LysZ5 produced a >4-log CFU/mL bacterial reduction in soya milk after 3 h of incubation at refrigerator temperature (4 °C) [50]. This was the first study to report that *Listeria* can be controlled in soya milk by endolysin treatment. *Listeria* has also been shown to be synergistically inactivated by a co-treatment of endolysin and high hydrostatic pressure, especially in pressure-sensitive foods (e.g., milk, mozzarella, and smoked salmon) [46]. Nisin has also shown a synergistic antilisterial effect with endolysin PlyP100 in Queso Fresco [48]. Furthermore, Ply511 and Ply118 have been shown to significantly reduce the number of viable *L. monocytogenes* cells when added to artificially spiked iceberg lettuce and whole cows’ milk [58]. Another endolysin, the *Streptococcus uberis* endolysin Ply700, effectively killed bacteria in milk (up to 81% were killed in 15 min) [59]. Finally, the streptococcal endolysin B30 and phage λSA2 showed a synergistic killing effect in cows’ milk and in the mastitis mouse model [52].

As detailed here, numerous studies describe the characterization of new endolysins with activities against foodborne pathogens—not only in vitro, but also when applied to food and utensils. Moreover, a few studies have revealed the safety of these endolysins in relation to human health [54,55]. Taken together, endolysins are promising biocontrol agents that have the potential to be used in a variety of raw and processed foods as well as in food-producing facilities.

## 6. Application of Endolysins against Biofilms for Surface Disinfection

Biofilms are sessile communities of microorganisms that grow on surfaces and are embedded in a self-produced extracellular matrix. They are composed of numerous bacterial cells attached on the surface and surrounded by an extracellular matrix containing a mixture of polysaccharides, proteins, and extracellular DNA. In the food industry, the control of bacterial biofilms is vital as their presence in the facilities or on the surfaces of utensils may cause serious harm to human health [60,61,62]. More worryingly, the bacteria embedded in biofilms are even more highly resistant to antibiotics or disinfectants than planktonic cells [63].

As alternatives to antibiotics, endolysins are also promising antibiofilm agents that can remove biofilms from food production environments. Various staphylococcal endolysins and their derivative proteins have shown strong biofilm removal activities against *S. aureus* and *Staphylococcus epidermidis*. For example, the staphylococcal endolysins Phi11 and SAP-2 removed biofilms formed on polystyrene surfaces [64,65], whereas the endolysin LysH5 also showed staphylococcal biofilm removal activities, with a lack of resistant cells following treatment [27]. In addition, the efficient bacterial removal activities of SAL200 endolysin have been revealed by safranin staining, cell reduction, and scanning electron microscopy [66]. Another staphylococcal endolysin, PlyGRCS, which contains a single EAD and kills MRSA, also disrupted biofilms [67]. Furthermore, CHAPk, which is a truncated form of LysK endolysin that only contains the N-terminal endopeptidase domain, can also remove *S. aureus* biofilms on surfaces [68]. In a recent study, the staphylococcal endolysin LysCSA13 effectively removed staphylococcal biofilms on various food contact surfaces, including polystyrene, glass, and stainless steel and reduced biofilm mass by approximately 80–90% [69]. In biofilms formed by other bacteria, the *Streptococcus suis* endolysin LysSMP was effective against 32 biofilm-forming strains and produced >80% disruption of biofilms relative to the reductions produced by antibiotics or phage only treatments [70]. The *Streptococcus pyogenes* endolysin PlyC was also revealed to rapidly degrade biofilm matrixes. Interestingly, although streptococcal cells within the biofilm become refractory to antibiotics, the biofilm matrix was rapidly destroyed by the PlyC treatment [71]. In other studies, the amidase domain of the *L. monocytogenes* phage vB_LmoS_293 endolysin inhibited biofilm formation on abiotic surfaces [72], whereas the *Salmonella* endolysin Lys68 in combination with malic or citric acid reduced biofilms by approximately 1-log CFU [32]. Finally, the *P. aeruginosa* endolysin LysPA26 reduced the number of viable counts of *P. aeruginosa* biofilm cells by 1- to 2-log CFU and destroyed the biofilm matrix [73].

Taken together, these studies suggest that endolysins are a promising anti-biofilm agent that could be used to reduce biofilm formation in the food industry. However, the biofilm removal activities of endolysin should be examined under more realistic conditions; specifically, flow cell-based models [74,75], multispecies biofilm matrixes [76,77], and surface coatings or substrates encountered in food processing facilities should be investigated [78].

## 7. Concluding Remarks

As this review shows, endolysins are promising new agents for the control of foodborne pathogens, particularly in food processing and preservation applications. Given their high host specificity, they can control only the targeted pathogens rather than the beneficial bacteria, such as probiotics, in foods. However, the application of endolysins should be carefully considered as their enzymatic properties can be changed under various physicochemical conditions such as temperature, pH, and NaCl concentrations. Endolysins can also prohibit the spread of antibiotic-resistant bacteria, which is a major problem worldwide. Since endolysins also have biofilm removal abilities, they could be applied to the surfaces of food-producing facilities. Although problems with endolysin application have previously existed, especially toward Gram-negative bacteria, various studies have now introduced novel strategies that utilize endolysins as control agents against Gram-negative pathogens. Therefore, endolysins are potentially powerful enzymes that could prevent foodborne infections and enhance safety in the field of food science.

## Figures and Tables

**Table 1:** Summary of the endolysins application against various foodborne pathogens in foods.

Target Host	Endolysin	Food Applications	Characteristics	Reference
*Staphylococcus aureus*	LysH5	Milk	About 8-log CFU/mL reduction at 37 °C in 6 h. Synergistic bactericidal effect with nisin.	[40,41]
	Ply187AN-KSH3b	Milk	About 3-log CFU/mL reduction at 37 °C immediately.	[42]
	λSA2-E-LysO-SH3b, λSA2-E-LysK-SH3b	Cow milk	About 3-log CFU/mL reduction at 37 °C in 3 h.	[43]
	HydH5Lyso, HydH5SH3b, CHAPSH3b	Milk	About 4-log CFU/mL reduction after CHAPSH3b treatment at 37 °C in 15 min.	[44]
	LysSA97	Milk, Beef	Synergistic bactericidal effect with carvacrol.	[45]
	LysSA11	Milk, Ham	About 4-log CFU/cm^3^ reduction at 25 °C in 15 min.	[17]
	Phi11-481 endolysin	Milk	Showed strong activity at 2–3 mM CaCl_2_.	[23]
*Listeria* *monocytogenes*	PlyP825	Milk Mozzarella	Synergistic bactericidal effect with high hydrostatic pressure.	[46]
	PlyP100	Cheese	About 3.5-log CFU/g reduction at 4 °C in 4 weeks.	[47]
		Queso Fresco	Synergistic bactericidal effect with nisin.	[48]
	Ply500	Iceberg lettuce	About 4-log CFU reduction at 25 °C in 24 h (free or immobilized endolysins).	[49]
	LysZ5	Soya milk	More than 4-log CFU/mL reduction in 3 h at 4 °C.	[50]
*Clostridium* *perfringens*	Ctp1L	Cow milk	About 1-log CFU/mL reduction in 2 h.	[51]
*Streptococcus* *dysgalactiae*	λSA2 lysin B30 lysin	Cow milk	Stronger activity with λSA2 lysin (3.5-log CFU/mL reduction at 100 μg/mL) than B30 lysin.	[52]
	ClyR	Milk	More than 2-log CFU/mL reduction within 1 min.	[53]
